# Large current difference in Au-coated vertical silicon nanowire electrode array with functionalization of peptides

**DOI:** 10.1186/1556-276X-8-502

**Published:** 2013-11-26

**Authors:** Ilsoo Kim, So-Eun Kim, Sanghun Han, Hyungsuk Kim, Jaehyung Lee, Du-Won Jeong, Ju-Jin Kim, Yong-beom Lim, Heon-Jin Choi

**Affiliations:** 1 Department of Materials Science and Engineering, Yonsei University, Seoul 120-749, Republic of Korea; 2 Department of Physics, Chonbuk National University, Jeonju 561-756, Republic of Korea

**Keywords:** Nanoelectrode, Silicon nanowire, Biomolecule sensing, Cyclic voltammograms, High current density

## Abstract

Au-coated vertical silicon nanowire electrode array (VSNEA) was fabricated using a combination of bottom-up and top-down approaches by chemical vapor deposition and complementary metal-oxide-semiconductor process for biomolecule sensing. To verify the feasibility for the detection of biomolecules, Au-coated VSNEA was functionalized using peptides having a fluorescent probe. Cyclic voltammograms of the peptide-functionalized Au-coated VSNEA show a steady-state electrochemical current behavior. Because of the critically small dimension and vertically aligned nature of VSNEA, the current density of Au-coated VSNEA was dramatically higher than that of Au film electrodes. Au-coated VSNEA further showed a large current difference with and without peptides that was nine times more than that of Au film electrodes. These results indicate that Au-coated VSENA is highly effective device to detect peptides compared to conventional thin-film electrodes. Au-coated VSNEA can also be used as a divergent biosensor platform in many applications.

## Background

Nanoelectrodes have many advantages such as a high current density and a large active area for chemical and biological sensing; the higher sensitivity of nanoelectrodes compared to bulk electrodes for the detection of various biological species has already been demonstrated [1-3]. Because of their unique shape with nanoscale diameters and micrometer-scale lengths that allow for an easy fabrication of device architectures, one-dimensional nanostructures, including carbon nanotubes and nanowires, are promising materials for nanoelectrodes [4-7]. Moreover, their possibility in highly complementary metal-oxide-semiconductor (CMOS) devices and their biocompatibility afford silicon nanowires (Si NWs) further advantages as nanoelectrodes [8-12].

Si NW sensors have been studied thus far with respect to applications as field effect-type transistor. These sensors are fabricated by first growing Si NWs on a substrate, dispersing them into solution such as deionized water or ethanol, depositing the nanowires on a pre-patterned substrate, and finally making metal contacts for electric current signaling. The functionalization of NWs with biological ligand molecules follows subsequently. In this type sensor, the electric field generated by the binding of charged biomolecules at the gate electrode acts as a gate voltage and changes the electric current, which, in turn, enables the sensing of biomolecules [13-15]. However, such sensors show low sensitivity because of the weak current difference induced by the low field effect of charged target biomolecules. These problems could be resolved by the electrochemical type sensor, especially using the vertical nanowire electrode array. In the electrochemical type sensor, the current occurs by the ion molecules in medium. In this situation, high current difference can be expected from the vertical nanowire electrode array when the biomolecules are attached at the nanosized electrode tip because the current path of the charged ion in a medium will be completely blocked. To exploit the potential of this approach, such vertical-type nanoelectrode array needs to be fabricated and biologically functionalized. In particular, the fabrication of such electrode array using a combination of a bottom-up approach that can provide the best suited bio-nanomaterials serving as building blocks of a sensor and a top-down approach that can provide a reliable device-fabrication process could be very useful for the mass production of high-performance biosensors for many applications.

In this paper, we fabricated Au-coated vertical Si nanowire electrode array (VSNEA) by combining a vapor-liquid-solid (VLS) process for the growth of nanowires and CMOS process for the fabrication of electrodes using these nanowires. The feasibility of biomolecule detection of such VSNEA was verified by functionalization with peptides having fluorescent probes and detecting the corresponding signals.

## Methods

### Growth of nanowires and fabrication of electrode array

Si (111) substrate was deposited by 0.1 vol.% 3-aminopropyl triethoxysilane (APTES) solution in absolute ethanol for Au colloid coating. Subsequently, the substrate was immersed in the Au colloid solution having colloids with a diameter of 250 nm. After washing with deionized water and drying, the substrates were placed in a low-pressure chemical vapor deposition (CVD) chamber. Si NWs were synthesized on the Si substrate by a VLS process with the assistance of the Au colloids serving as catalyst at 550°C under a high-vacuum condition with SiH_4_ gas as a precursor and H_2_ as a dilution gas. For the fabrication of the electrodes, Si NWs grown on the substrate were coated with an Au electrode and a SiO_2_ passivation layer. Thereafter, the SiO_2_ passivation layer was selectively etched out to expose the Au tips at the top of the nanowires by using CMOS process.

### Synthesis of peptides

Peptides were synthesized on Rink Amide MBHA resin LL (Novabiochem, Merck KGaA, Darmstadt, Germany) using standard Fmoc protocols on a Tribute™ peptide synthesizer (Protein Technologies, Inc, Tucson, AZ, USA). All amino acids were purchased from Novabiochem. 5(6)-carboxy fluorescein was purchased from Sigma-Aldrich (St. Louis, MO, USA). Fluorescein was attached to the peptide-attached resin (10 μmol of N-terminal amine groups). A mixture of 5(6)-carboxyfluorescein (19 mg, 50 μmol), 2-(1H-benzotriazole-1-yl)-1,1,3,3-tetramethyluronium hexafluorophosphate (HBTU) (18 mg, 47.5 μmol), and N,N-diisopropylethylamine (DIPEA) (20 μL, 115 μmol) in N-methyl-2-pyrrolidone (NMP) was incubated for 10 min for carboxyl activation. The resulting solution was mixed with the resin and the reaction was continued for 18 h with shaking at room temperature in a 15-mL conical tube. Then, the resin was washed with tetrahydrofuran (THF) and blown dry with high-purity argon gas. The dried resin was treated with a cleavage cocktail (trifluoroacetic acid (TFA)/1,2-ethanedithiol/thioanisole 95:2.5:2.5) for 3 h and was triturated with tert-butyl methyl ether. The peptides were purified by reverse-phase high-performance liquid chromatography (HPLC) (water-acetonitrile with 0.1% of TFA). The molecular weight was confirmed by MALDI-TOF mass spectrometry. The concentration was determined spectrophotometrically using the molar extinction coefficient of fluorescein (65,000 M-1 cm-1) at 492 nm.

### Peptide functionalization of VSNEA

Au-coated VSNEA was immersed in a tissue culture well (BD Falcon™ 24-well Multiwell Plate, BD Biosciences, San Jose, CA, USA) and a mixture of peptides (5 μL of 2.13 μM) and water (700 μL) was added. The reaction was continued for 12 h. Afterward, the substrate was washed multiple times with distilled water and dried in air.

### Microscopy and CV measurements

Peptide-decorated Au-coated VSNEA was observed by bright field and fluorescence microscopy (Olympus BX51, U-HGLGPS, Olympus Corporation, Shinjuku, Tokyo, Japan), as well as by scanning and transmission electron microscopies (SEM and TEM, respectively). For the electrochemical characterization of VSNEA, cyclic voltammograms (CV) measurements were carried out using an IVIUM CompactStat system in standard three-electrode configuration. VSNEA, an Au film electrode, and Ag/AgCl were used as working, counter, and reference electrodes, respectively, of the three-electrode configuration. CVs were performed with a 100-mM K_3_Fe(CN)_6_ solution as redox material at a scan rate of 20 mV/s.

## Results and discussion

In this study, vertically grown Si NWs were used as building blocks for Au-coated VSNEA. It requires vertical growth, as well as control of the areal density of NWs achieved by applying CMOS processing for the electrodes, thereby additionally performing a detection optimization. To control the growth of Si NWs, we utilized a VLS process using Au colloidal nanoparticles as catalyst [16,17]. To thoroughly disperse the Au nanoparticles, Si substrate was coated with a thin 3-aminopropyl triethoxysilane (APTES) layer. It is well known that Au nanoparticles have a negatively charged surface in aqueous solution and APTES has a positively charged functional amino group [18]. Therefore, the charged surface of the Si substrate deposited with an APTES layer can be used to establish a charge interaction with the Au nanoparticles, thus enabling to achieve a fine and homogeneous dispersion of 250-nm Au nanoparticles on the Si (111) substrate (See Additional file 1: Figure S1 of supplementary data).

**Figure 1:**
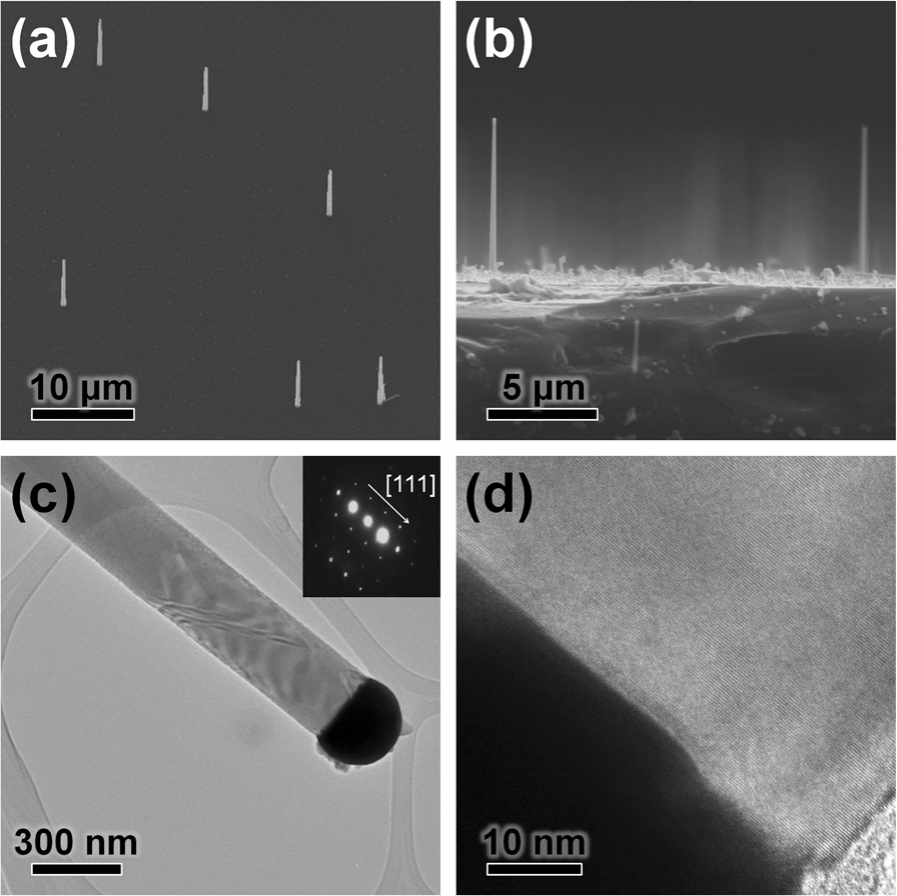
**SEM and TEM images of the synthesized Si NWs. (a)** SEM image tilted by 30° and **(b)** cross-sectional SEM image of vertical Si NWs. **(c)** Typical low-magnitude TEM image and **(d)** HRTEM image of a Si NW. The inset of part (c) shows a SAED pattern of the NW. The inset SAED pattern is related with a crystal plane of the NW, and the NW growth direction spots express the [111] crystal planes. It indicates Si NW has [111] growth direction.

Figure 1a,b shows an SEM image of the Si NWs, indicating that they are well dispersed and grown vertically on the Si (111) substrate. The length of the NWs is approximately 8 μm. Figure 1c is the TEM image of the Si NWs. As shown in this figure, the diameter of the Si NWs was approximately 300 nm, thus following the size of the Au nanoparticles used as catalyst in the VLS process (See Additional file 1: Figure S2 of supplementary data). The selected area electron diffraction (SAED) pattern presented in the inset of Figure 1c indicates that the Si NWs are single crystalline and grew along the [111] direction. Figure 1d, a high-resolution transmission electron microscopy (HRTEM) image, also shows the single-crystalline nature of the Si NWs with a thin native oxide layer with a thickness of less than 2 nm. The metal globule at the end of NWs clearly indicates that the Si NWs were synthesized by the VLS mechanism. These results indicate that vertical Si NWs are epitaxially grown on the Si (111) substrate with a single-crystalline structure, and their NWs density can be well controlled by controlling the concentration of Au nanoparticles.

**Figure 2:**
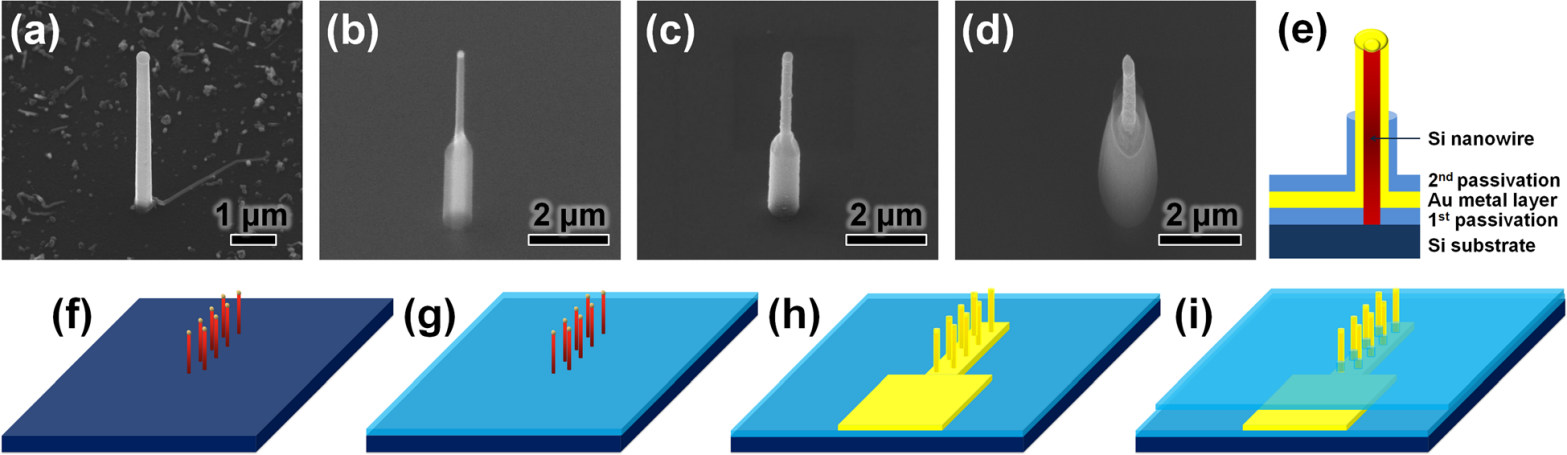
**Fabrication scheme of Au-coated VSNEA and corresponding SEM images. (a to i)** SEM images tilted by 30^o^. **(a, f)** Vertical Si NWs grown on a (111) Si substrate; **(b, g)** Si NW array coated with a first SiO_2_ passivation layer; **(c, h)** Si NW array after coating with an Au electrode; **(d, i)** Si NW electrode array coated with a second SiO_2_ passivation layer. **(e)** Cross-sectional scheme of VSNEA.

Figure 2 shows the scheme of the Au-coated VSNEA fabrication procedure with vertical Si NWs and the corresponding SEM images of the individual fabrication steps. A Si NW array was grown on the Si (111) substrate (shown in Figure 2a,f), and a first SiO_2_ passivation layer was coated on the surface by chemical vapor deposition (CVD) process. The SiO_2_ layer was then selectively etched to expose the Si NWs, as shown in Figure 2b,g. For this purpose, poly (methyl methacrylate) (PMMA) resist serving as a SiO_2_ protecting layer was coated on the substrate and the side walls of the NWs were selectively etched by dipping them into buffered oxide etch (BOE). Next, an Au film was deposited on the surface of the NWs to attach the peptides that can induce biological functionalization, as well as to act as an electrode within a sensing device, as shown in Figure 2c,h. A 50-nm-thick Au film was deposited by a sputtering process using a patterned steel use stainless (SUS) mask at a slow rate of 5 nm per minute to avoid roughening of the surface or thermal damage under an argon atmosphere (shown in Figure 2c,h). Finally, the same type of passivation using a SiO_2_ layer was performed once more (shown in Figure 2d) by CVD process. The secondary SiO_2_ passivation layer was then selectively etched to expose the Au layer only at the tip of VSNEA, as shown in Figure 2d,i. Figure 2e shows a cross-sectional scheme of the finished Au-coated VSNEA.

To verify the feasibility of Au-coated VSNEA for biomolecules detection, peptides having a fluorescent probe, carboxyfluorescein, were attached the surface on NWs. Peptides are well suited as the bioreceptor component of various biosensors and biomedical applications because they can selectively and tightly bind to a large diversity of biomolecules, including DNA, RNA, and protein targets through modification of their multi-linking polychains [19,20]. It is also well known that sulfur makes a strong covalent bond with Au in a wide range of temperatures for a variety of solvents [21]. Therefore, a 13-mer peptide was synthesized using solid phase peptide synthesis (SPPS) with standard Fmoc protocols. The peptide was designed to incorporate multiple polar and charged residues (glutamic acids and lysines) in order to increase its water solubility and to prevent nonspecific adsorption of the peptide onto the VSNEA specimen.

**Figure 3:**
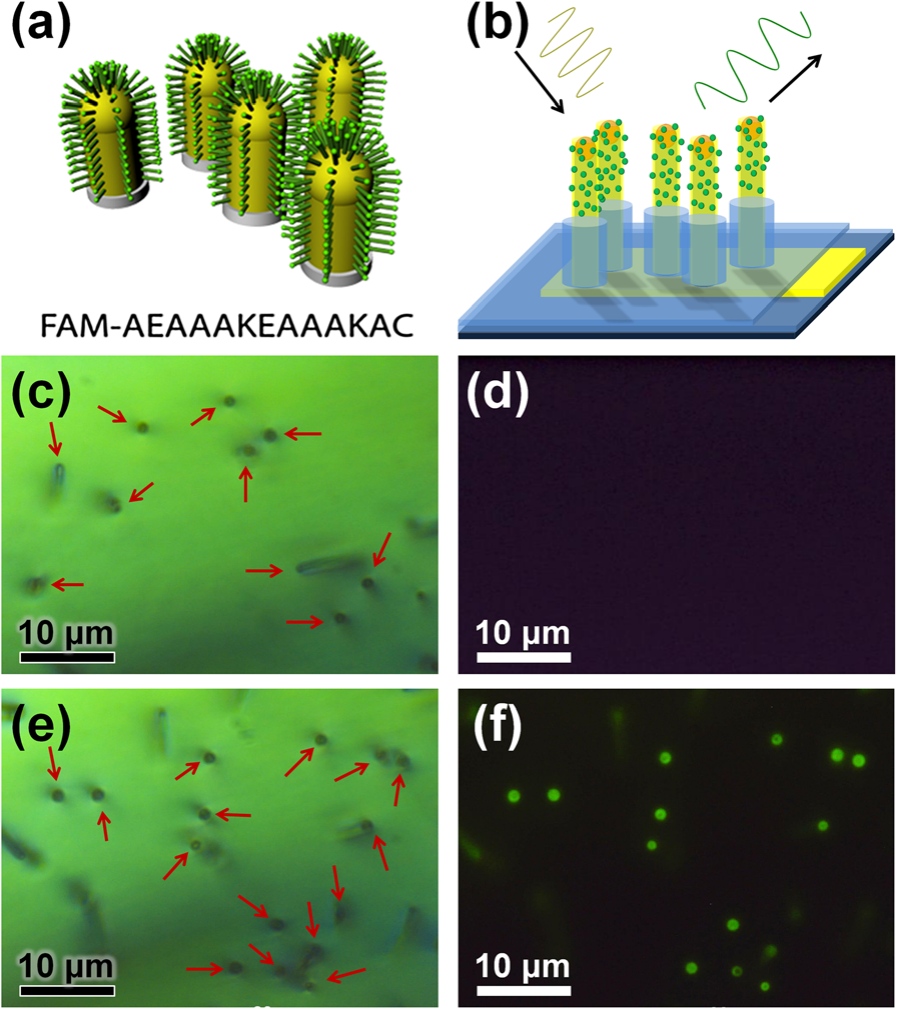
**Scheme of peptide-functionalized Au-coated NWs and bright field and fluorescence microscopy images. (a)** Sequence of peptides labeled with carboxyfluorescein and schematic model of peptide-decorated Au-coated vertical Si NWs. **(b)** Scheme for fluorescence analysis of peptide-decorated Au-coated VSNEA. Bright field image **(c)** and corresponding fluorescence image **(d)** of the non-decorated VSNEA. (Black dots indicated with red arrows represent vertical NWs). Bright field image **(e)** and corresponding fluorescence image **(f)** of the peptide-decorated VSNEA (black dots indicated by red arrows again represent vertical NWs). All microscopy images are taken at a magnification of 1,000.

A fluorescent probe, carboxyfluorescein, was attached to the N-terminus of the peptide to visualize the peptide binding to the NWs in a noninvasive manner (Figure 3a). Cysteine that contains a thiol group was placed at the C-terminal of the peptide for the formation of thiol-Au bond. To verify that the peptides bind selectively to the Au tips of VSNEA, they were immersed in an aqueous solution of the peptide, incubated for 12 h, and subsequently washed thoroughly with distilled water. The number of peptide molecules in the solution was in large excess relative to the calculated surface area of the Au tips.

Investigations by bright field and fluorescence microscopy were carried out to confirm that peptides were selectively attached to the active Au tip of VSNEA. Figure 3b shows the scheme for the fluorescence analysis of the peptide-decorated Au-coated VSNEA. As shown in Figure 3c, the vertical NWs are observed in the form of small dots in the bright field microscope image. Because this VSNEA specimen has not been treated with peptides, its fluorescence image did not reveal any sign of green fluorescence originating from the fluorescein molecules (Figure 3d). In stark contrast, the VSNEA specimen treated with peptides shows bright green fluorescence light of fluorescein (Figure 3e,f). Colocalization of dots in the upright microscopy images of VSNEA, as evidenced by a comparison between the bright field and the corresponding fluorescence images, strongly suggests a selective binding of the peptides to the Au tips of VSNEA without any nonspecific binding to other areas of the substrate (Figure 3a,b). Calculations considering the volume of the peptide molecule and the surface area of Au show that multiple peptide molecules can be attached to a single Au tip. Such multiple additions of peptides onto Au-coated VSNEA are expected to be useful in exploring biological multivalent interactions.

**Figure 4:**
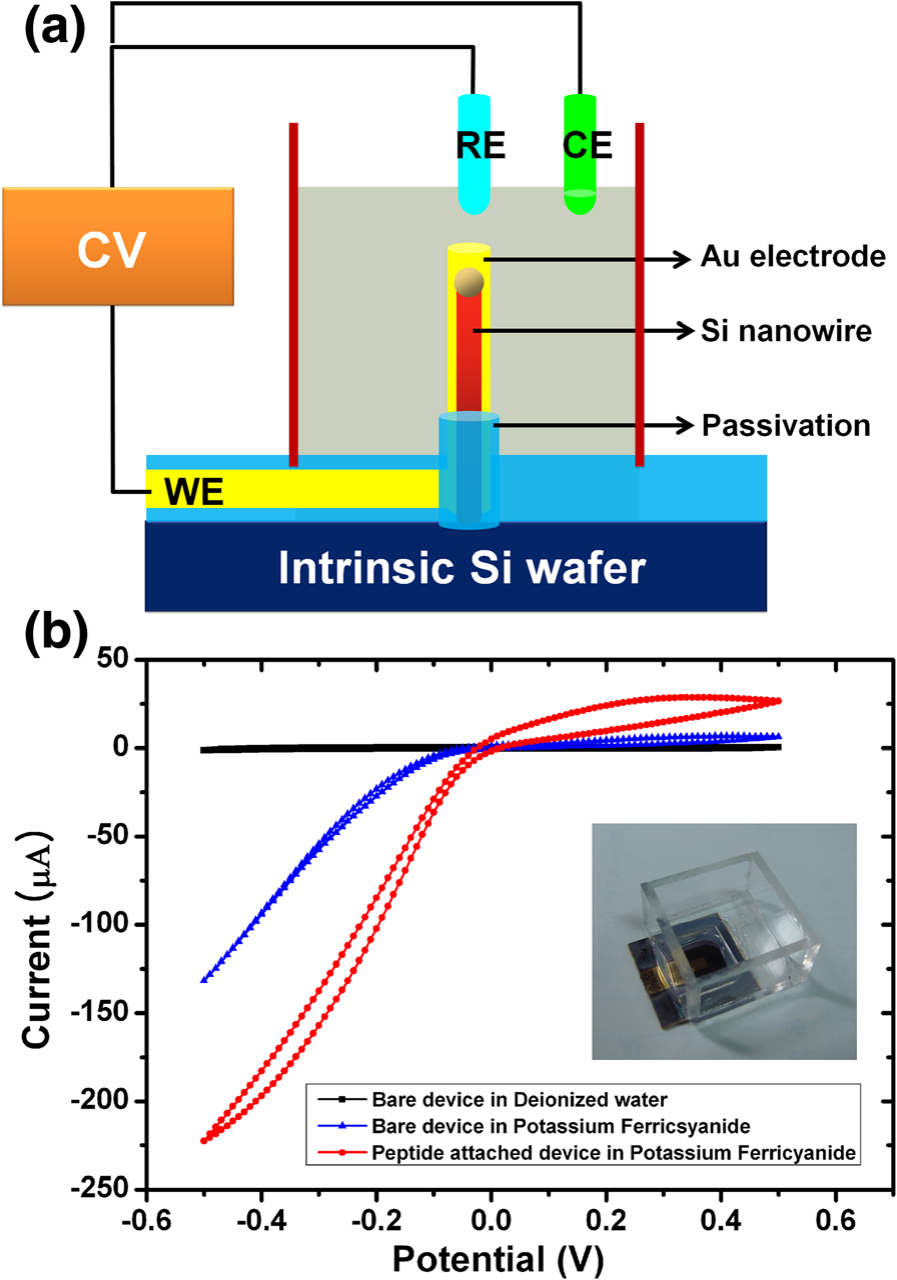
**Schematic of three-electrode configuration system and CV measurements of Au-coated VSNEA. (a)** Schematic of three-electrode configuration used in CV measurement system with Au-coated VSNEA as a working electrode. **(b)** CV measurements of Au-coated VSNEA in deionized water and 100 mM K_3_Fe(CN)_6_. All CV measurements are performed at a scan rate of 20 mV/s. The inset of part (b) shows an electrolyte bath attached the VSNEA device for CV measurements.

Au-coated VSNEA was further characterized by cyclic voltammogram (CV) measurements. Figure 4a,b shows an electrolyte bath attached VSNEA device for CV measurements and the schematic of the three-electrode configuration of the CV measurement system using Au-coated VSNEA as a working electrode. Figure 4b and Additional file 1: Figure S3 (See Additional file 1: Figure S3 of supplementary data) show the CV data of Au-coated VSNEA and an Au film device, respectively. The Au film device was prepared by coating of 50-nm-thick Au on a flat Si substrate and characterized under identical conditions for comparison. As in the case of many other electrochemical nanoelectrodes, the CV of VSNEA shows a steady-state electrochemical current behavior caused by an enhanced mass transport and fast electron-transfer kinetics owing to its nanosize and unique shape [4-7,22,23].

The detection difference with respect to biomolecules was analyzed by CV measurements of the VSNEA and the results are summarized in Tables 1 and 2. Here, *Q*_bare_ is the coulomb of the electrode before peptide functionalization, *Q*_peptide_ is the coulomb of the peptide-decorated electrode, *A* is the total electrode area, and *J* is the current density of the electrode. As shown in Table 1, the current density of Au-coated VSNEA was approximately 7,000 times higher than that of an Au film electrode prior to functionalization by peptides, whereas after peptides decoration, the current density of the VSNEA was still approximately 3,000 times higher than that of the peptide-decorated Au film electrode. The much higher current density of Au-coated VSNEA is because of the critically small dimension and the vertically aligned nature of the NWs affecting the mass transport and the electron transfer. The mass transport and electron concentration of Au-coated VSNEA electrode are higher than that in the case of an Au film electrode because of the cylindrical shape of the nanowire (See Additional file 1: Figure S4 of supplementary data).

**Table 1:** Calculation of current density of Au film and VSNEA based on CV measurements

**Au film**		**VSNEA**
*Q*_bare*,*film_ = 1.636 × 10^-2^*C*		*Q*_bare*,*NWs_ = 1.163 × 10^-1^*C*
*Q*_peptide*,*film_ = 1.529 × 10^-2^*C*		*Q*_peptide*,*NWs_ = 4.899 × 10^-2^*C*
*A*_film_ = 4.9 × 10^-5^ *m*^2^		*A*_NWs_ = 4.925 × 10^-8^ *m*^2^
$\begin{array}{l} J_{\mathrm{bare},\mathrm{film}}=\frac{Q_{\mathrm{bare},\mathrm{film}}}{A_{\mathrm{film}}\times 1s}\\ \begin{array}{l} \;=\frac{1.636\times 10^{-2}\;C}{4.9\times 10^{-5}\;m^{2}\times 1s}\\ \;=3.339\times 10^{2}A/m^{2} \end{array} \end{array}$		$\begin{array}{l} J_{\mathrm{bare},\mathrm{NWs}}=\frac{Q_{\mathrm{bare},\mathrm{NWs}}}{A_{\mathrm{NWs}}\times 1s}\\ \begin{array}{l} \;=\frac{1.163\times 10^{-1}\;C}{4.925\times 10^{-8}\;m^{2}\times 1s}\\ \;=2.361\times 10^{6}A/m^{2} \end{array} \end{array}$
	$\frac{J_{\mathrm{bare},\mathrm{NWs}}}{J_{\mathrm{bare},\mathrm{film}}}=\frac{2.361\times 10^{6}\;A/m^{2}}{3.339\times 10^{2}\;A/m^{2}}=7071$	
$\begin{array}{l} J_{\mathrm{peptide},\mathrm{film}}=\frac{Q_{\mathrm{peptide},\mathrm{film}}}{A_{\mathrm{film}}\times 1s}\\ \begin{array}{l} \;=\frac{1.529\times 10^{-2}\;C}{4.9\times 10^{-5}\;m^{2}\times 1s}\\ \;=3.120\times 10^{2}A/m^{2} \end{array} \end{array}$		$\begin{array}{l} J_{\mathrm{peptide},\mathrm{NWs}}=\frac{Q_{\mathrm{peptide},\mathrm{NWs}}}{A_{\mathrm{NWs}}\times 1s}\\ \begin{array}{l} \;=\frac{4.899\times 10^{-2}\;C}{4.925\times 10^{-8}\;m^{2}\times 1s}\\ \;=9.947\times 10^{5}A/m^{2} \end{array} \end{array}$
	$\frac{J_{\mathrm{peptide},\mathrm{NWs}}}{J_{\mathrm{peptide},\mathrm{film}}}=\frac{9.947\times 10^{5}A/m^{2}}{3.120\times 10^{2}A/m^{2}}=3188$	

**Table 2:** Calculation of current difference of a Au film and a VSNEA electrode, based on the results of CV measurements

**Au film**		**VSNEA**
$\begin{array}{l} \delta_{\mathrm{film}}=\frac{Q_{\mathrm{peptide},\mathrm{flim}}-Q_{\mathrm{bare},\mathrm{film}}}{Q_{\mathrm{bare},\mathrm{film}}}\times 100\\ \;=\frac{1.529\times 10^{-2}\;C-1.636\times 10^{-2}\;C}{1.636\times 10^{-2}\;C}\times 100\\ \;=-6.540\;\% \end{array}$		$\begin{array}{l} \delta_{\mathrm{NWs}}=\frac{Q_{\mathrm{peptide},\mathrm{NWs}}-Q_{\mathrm{bare},\mathrm{NWs}}}{Q_{\mathrm{bare},\mathrm{NWs}}}\times 100\\ \;=\frac{4.899\times 10^{-2}\;C-1.163\times 10^{-1}\;C}{1.163\times 10^{-1}\;C}\times 100\\ \;=-57.876\;\% \end{array}$
	$\frac{\delta_{\mathrm{NWs}}}{\delta_{\mathrm{film}}}=\frac{-57.876\;\%}{-6.540\;\%}=8.850$	

Au-coated VESNA showed large differences in the current with and without the functionalization of peptides. As shown in Table 2, the current of Au-coated VSNEA with peptides decreased by 57.8% compared to the VSNEA without peptides. This large current difference (*δ*) was almost nine times more than that of the Au film electrodes. The results indicate that the two electrochemical type sensors can detect the target molecules by a current difference that is ascribed to the flow variations of ion molecules in medium. Furthermore, the results represent that the current path through the Au nanoelectrodes in the electrochemical type VSNEA is effectively blocked by the attached peptides and thus it can effectively detects the peptides. The high current density of VSNEA also ascribe to the large difference in current with and without peptides. These outcomes indicate that VSNEA can detect peptides better than film-type electrodes. It should be noted that peptides can be used as ligands to detect various other biomolecules and thus VSNEA could be used as divergent biosensor platforms in many applications.

## Conclusions

We vertically grew Si NWs and fabricated Au-coated VSNEA for the peptide detection. The VSNEA was selectively functionalized by multiple peptides to verify their application potential for biomolecules detection. We obtain a steady-state electrochemical current behavior and a high current density from peptides-functionalized Au-coated VSNEA because of the critically small dimension and the vertically aligned nature of this device. Furthermore, VSNEA showed a large current difference with and without peptides that was nine times more than that of Au film electrodes. These results indicate that VSNEA is highly effective to detect peptides, compared to conventional thin-film electrodes. Therefore, VSNEA could be used as divergent biosensor platforms in many applications.

## Competing interests

The authors declare that they have no competing interests.

## Authors’ contributions

IK and SEK carried out the experiment and drafted the manuscript. SH, HK, JL, DWJ, JJK, YL, and HJC participated in the design of the study and drafted the manuscript. All authors read and approved the final manuscript.

## Supplementary Material

Additional file 1: Figure S1 SEM images of 250 nm Au nanoparticles dispersed on the Si substrates. (a, b) 1 (Au nanoparticles) : 3 (DI water) solution. (c, d) 1 (Au nanoparticles) : 6 (DI water) solution. **Figure S2**. Scanning TEM images of the Si NW. (a) Energy dispersive scanning point of the Si NW. (b) Energy dispersive spectrum for each scanning point. **Figure S3**. (a) Schematic image of CV measurements system using Au film as a working electrode. (b) CV measurements of the Au film electrode in 100 mM K_3_Fe(CN)_6_. All CV measurements are taken with a scan rate of 20 mV/s. **Figure S4**. Schematic images of ion and electron transfer mechanism in the CV measurements system using Au film (a, b) and VSNEA (c, d) as a working electrode. Click here for file
