# Supplementary material for: Large current difference in Au-coated vertical silicon nanowire electrode array with functionalization of peptides
Source: Nanoscale Res Lett. 2013 Nov 26;8(1):502. doi: 10.1186/1556-276X-8-502 (PMC4221645; doi:10.1186/1556-276X-8-502)

**Electronic supplementary data for Nanoscale Research Letters**

**Electronic Supporting Information**

**Highly sensitive detection of peptides by using vertical silicon nanowire electrode array**

Ilsoo Kim^1^Email: nicepolo@yonsei.ac.kr

So-Eun Kim^1^Email: k.ssogood@gmail.com

Sanghun Han^1^Email: yamehand@gmail.com

Du-Won Jeong^2^Email: dowone@jbnu.ac.kr

Ju-Jin Kim^2^Email: jujinkim@jbnu.ac.kr

Yong-beom Lim^1,^*
* Corresponding author
Email: yblim@yonsei.ac.kr

Heon-Jin Choi^1,^*
* Corresponding author
Email: hjc@yonsei.ac.kr

^1^ Department of Materials Science and Engineering, Yonsei University, Seoul 120-749, Republic of Korea.

^2^ Department of Physics, Chonbuk National University, Jeonju 561-756, Republic of Korea.

**Figure S1.** SEM images of 250 nm Au nanoparticles dispersed on the Si substrates.
(a, b) 1 (Au nanoparticles) : 3 (DI water) solution. (c, d) 1 (Au nanoparticles) : 6 (DI water) solution.


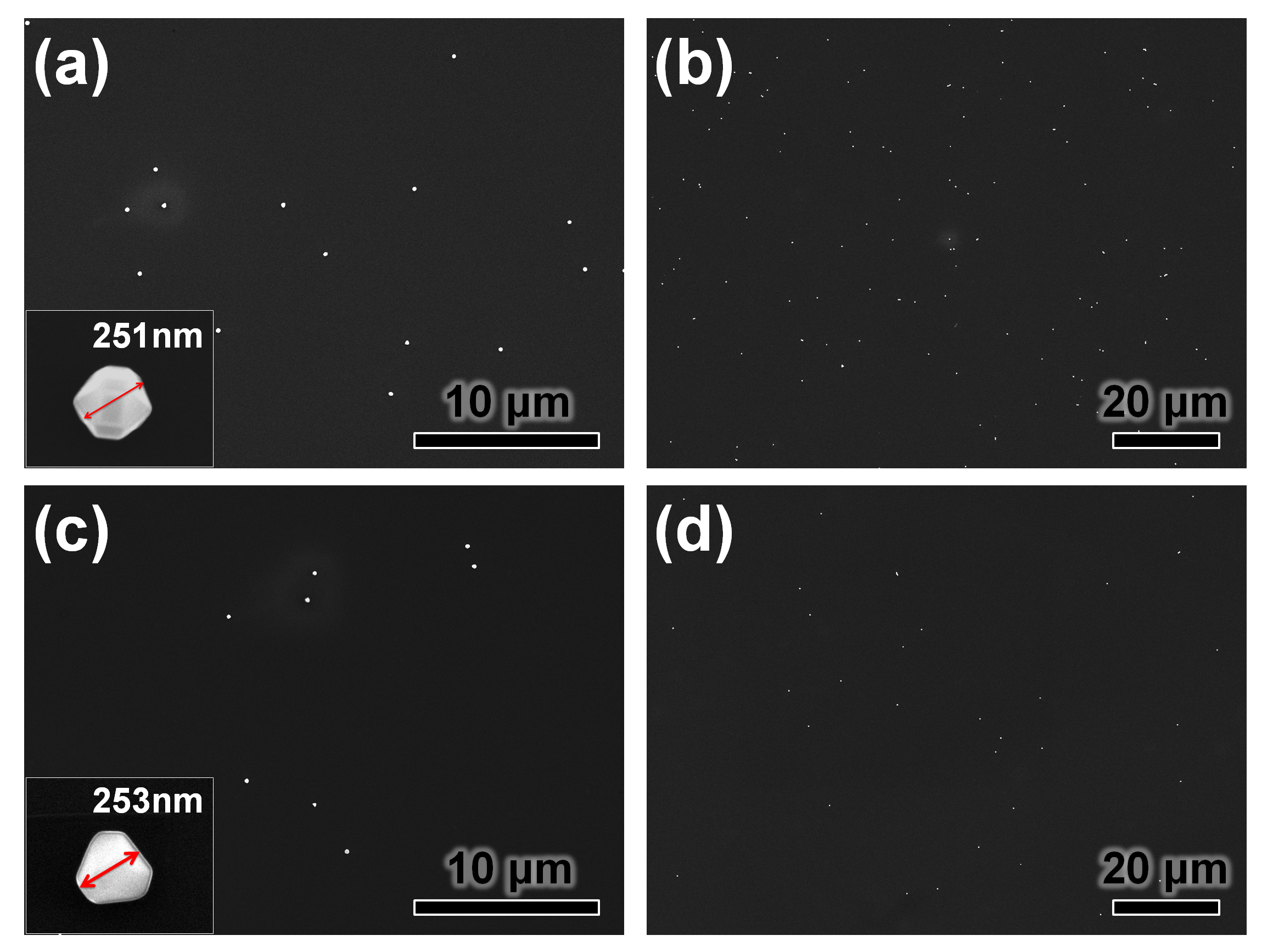


**Figure S2.** Scanning TEM images of the Si NW. (a) Energy dispersive scanning point of the Si NW. (b) Energy dispersive spectrum for each scanning point.


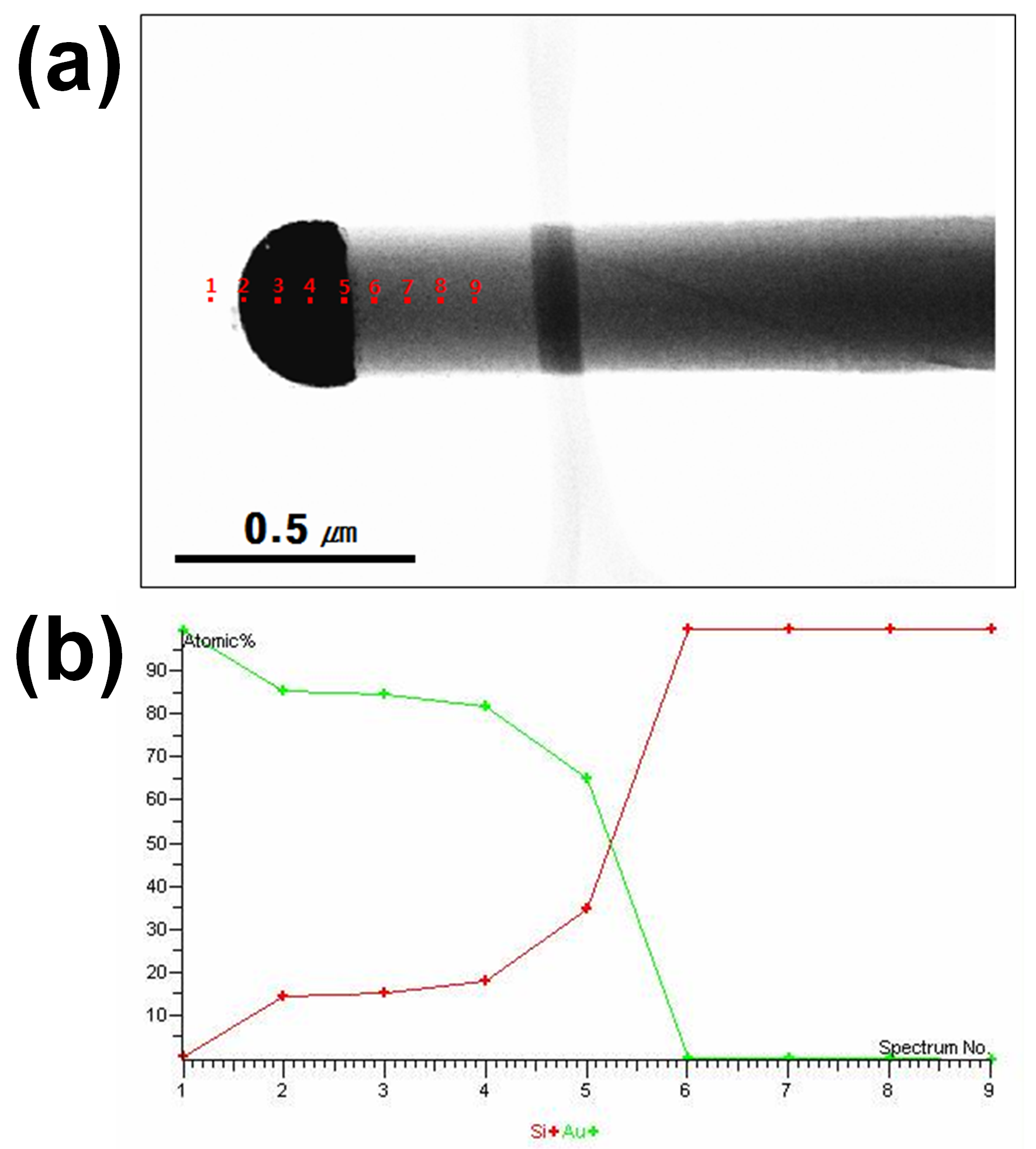


**Figure S3.** (a) Schematic image of CV measurements system using Au film as a working electrode. (b) CV measurements of the Au film electrode in 100mM K_3_Fe(CN)_6_. All CV measurements are taken with a scan rate of 20 mV/s.


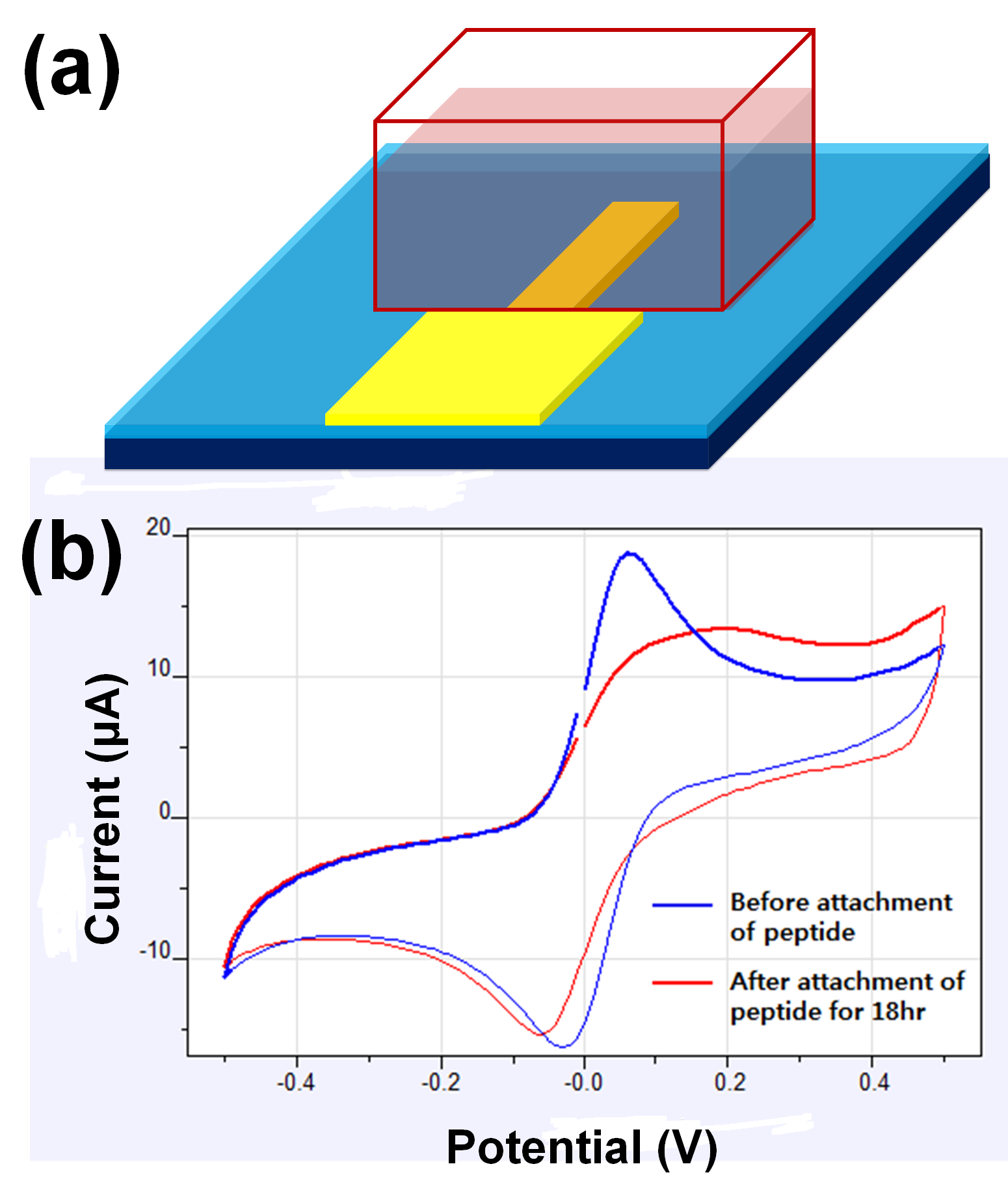


**Figure S4.** Schematic images of ion and electron transfer mechanism in the CV measurements system using Au film (a, b) and VSNEA (c, d) as a working electrode.


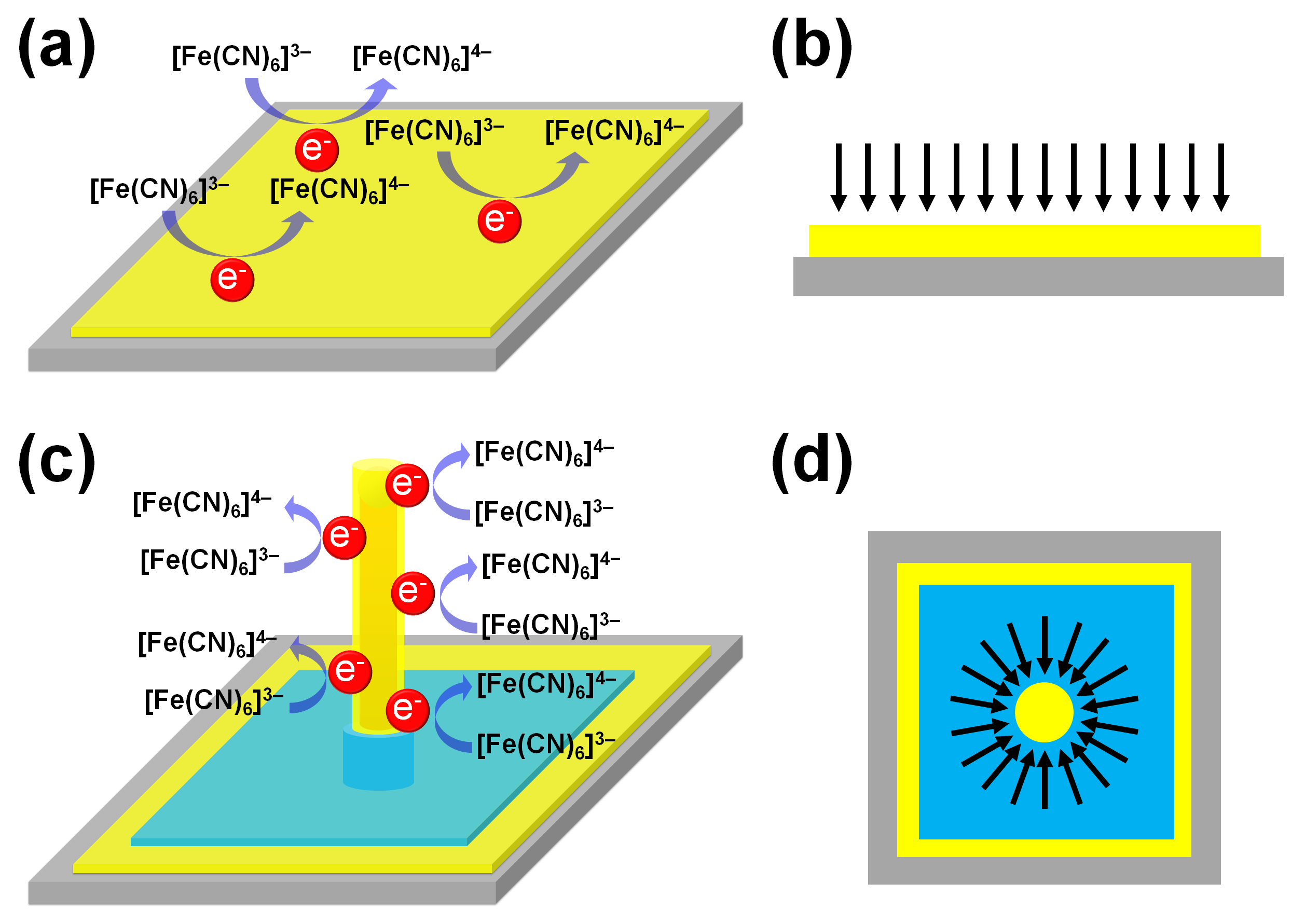

Supplement: Additional file 1: Figure S1 — SEM images of 250 nm Au nanoparticles dispersed on the Si substrates. (a, b) 1 (Au nanoparticles) : 3 (DI water) solution. (c, d) 1 (Au nanoparticles) : 6 (DI water) solution. Figure S2. Scanning TEM images of the Si NW. (a) Energy dispersive scanning point of the Si NW. (b) Energy dispersive spectrum for each scanning point. Figure S3. (a) Schematic image of CV measurements system using Au film as a working electrode. (b) CV measurements of the Au film electrode in 100 mM K3Fe(CN)6. All CV measurements are taken with a scan rate of 20 mV/s. Figure S4. Schematic images of ion and electron transfer mechanism in the CV measurements system using Au film (a, b) and VSNEA (c, d) as a working electrode. [file 1556-276X-8-502-S1.docx]
